# Introduction to ‘Recent progress and open frontiers in Turing’s theory of morphogenesis’

**DOI:** 10.1098/rsta.2020.0280

**Published:** 2021-12-27

**Authors:** Andrew L. Krause, Eamonn A Gaffney, Philip K. Maini, Václav Klika

**Affiliations:** ^1^ Wolfson Centre for Mathematical Biology, Mathematical Institute, University of Oxford, Andrew Wiles Building, Radcliffe Observatory Quarter, Woodstock Road, Oxford OX2 6GG, UK; ^2^ Department of Mathematical Sciences, Durham University, Upper Mountjoy Campus, Stockton Rd, Durham DH1 3LE, UK; ^3^ Department of Mathematics, FNSPE, Czech Technical University in Prague, Trojanova, 13, 120 00 Praha, Czech Republic

**Keywords:** Turing instabilities, pattern formation, reaction-diffusion systems

## Abstract

Elucidating pattern forming processes is an important problem in the physical, chemical and biological sciences. Turing's contribution, after being initially neglected, eventually catalysed a huge amount of work from mathematicians, physicists, chemists and biologists aimed towards understanding how steady spatial patterns can emerge from homogeneous chemical mixtures due to the reaction and diffusion of different chemical species. While this theory has been developed mathematically and investigated experimentally for over half a century, many questions still remain unresolved. This theme issue places Turing's theory of pattern formation in a modern context, discussing the current frontiers in foundational aspects of pattern formation in reaction-diffusion and related systems. It highlights ongoing work in chemical, synthetic and developmental settings which is helping to elucidate how important Turing's mechanism is for real morphogenesis, while highlighting gaps that remain in matching theory to reality. The theme issue also surveys a variety of recent mathematical research pushing the boundaries of Turing's original theory to more realistic and complicated settings, as well as discussing open theoretical challenges in the analysis of such models. It aims to consolidate current research frontiers and highlight some of the most promising future directions.

This article is part of the theme issue ‘Recent progress and open frontiers in Turing’s theory of morphogenesis’.

The perspective of the scientific community on Turing’s theory of morphogenesis has undergone enormous changes since its inception almost 70 years ago [1]. It was mostly neglected, with only a few critical papers discussing it in the decades following the publication, in 1952, of *The chemical basis of morphogenesis* [2], though interest from both mathematicians and biologists increased in the 1970s following preliminary investigations of possible biological morphogens, as well as the increased use of computers to visualize Turing’s predicted patterns. In the late 1980s and early 1990s, chemical experiments demonstrated this pattern-forming mechanism in a laboratory setting [3]. The past two decades have seen an increase in mathematical, chemical and biological work extending, or relating to, Turing’s ideas on morphogenesis.

Progress has been made in corroborating the theory against biological data, extending the mathematical theory in several distinct directions, and using the Turing framework as a paradigm for pattern formation in a number of specific scenarios. This theme issue reviews and contributes to these areas broadly, and maps out future directions and the challenges remaining. While we will primarily focus on patterning processes related to Turing instabilities in reaction–diffusion systems, several of the contributions go beyond this framework and discuss broader classes of models, and related branches of theoretical and experimental exploration. Contemporary advances in experimental techniques, from modern microscopy to genetic perturbations, allow for much more precise experimental observations, but the classical theoretical frameworks of pattern formation are difficult to use to interpret such information directly. Novel perspectives on pattern forming systems are needed to help conceptualize these data, together with putting forward and testing concrete mechanistic hypotheses underlying biological morphogenesis.

Skin patterns on zebrafish have been studied intensively for many years, as a paradigmatic model of biological pattern formation. Kondo *et al*. [4] review this subject, showing that while observations are consistent with aspects of classical Turing pattern formation in reaction–diffusion systems (e.g. short-range activation and long-range inhibition), these mechanisms are insufficient to explain biological observations alone. Studies adapting Turing’s classical model to include cell migration and proliferation, as well as non-local interactions due to cell protrusions, are discussed in detail. Painter *et al*. [5] review a broader class of taxa, discussing periodic patterns in vertebrates. They reference a compelling body of work strongly supporting short-range activation and long-range inhibition mechanisms underlying periodic spacing and initial symmetry breaking in developing vertebrate skin, while also suggesting that more work is needed in understanding cellular and mechanical aspects of these pattern-forming processes. They make a number of important points in discussing the evidence so far, such as suggesting the consideration of more complicated explanations for developmental structure. Such explanations include the coexistence of multiple pattern-forming mechanisms, or hierarchical arrangements thereof. They highlight a range of specific near-term ideas to pursue, such as considering combined reaction-diffusion and chemotaxis paradigms in planar skin patterning, as well as the promising exploration of three-dimensional branching structures in vertebrate organ systems.

In a similar spirit of extending Turing’s insights, Krause *et al.* [6] review a wide range of the literature on near-equilibrium analysis of reaction–diffusion and related systems. They discuss models where a classical Turing-type analysis can be carried out with only minor modifications, such as in reaction–diffusion systems on curved manifolds or spatial networks, as well as cases where very different approaches are needed for even linear stability analysis, such as in heterogeneous systems. The review highlights a variety of mathematical challenges throughout, and the importance of aligning such problems with experimental observations. The authors suggest that the development of theoretical tools can be invaluable in aiding the understanding of contemporary biological observations. Van Gorder [7] provides an example of such a tool, exploiting a Galerkin expansion of heterogeneous reaction–diffusion systems in order to determine conditions under which a heterogeneous steady state is linearly unstable to perturbations, generalizing the classical Turing instability. Additionally, Van Gorder uses the evolution of nonlinear modes (from a suitable truncated system) in order to understand the competition and interaction of distinct modes, mediated via the spatial heterogeneity in the system. A range of examples are provided to demonstrate this theory, and to show how spatial heterogeneity can influence pattern formation in reaction–diffusion systems in a variety of different ways.

Al Saadi & Champneys [8] provide another tool for understanding dynamics of reaction-diffusion systems. Building on past work, they use spatial dynamics, weakly nonlinear analysis, and numerical continuation to find a general type of bifurcation diagram in a class of reaction–diffusion systems interpolating between qualitatively different kinds of reaction kinetics (e.g. monostable and bistable systems). Their bifurcation results indicate a number of relationships between classical Turing and Hopf bifurcations, with more exotic structures such as homoclinic snaking, as well as a variety of codimension-2 bifurcations. They conjecture a universality in the bifurcation regimes found, and give important arguments as to how such information can be valuable in modelling natural phenomena. Whereas Al Saadi and Champneys start with particular classes of models in mind, Gandhi *et al.* [9] take the opposite viewpoint and consider the problem of identifying and quantifying symmetry in natural systems in the absence of an underlying mechanistic model. Based on information-theoretic considerations from condensed-matter physics, they propose a suite of measures of symmetry with the aim of providing a universal quantitative operationalization of the term across a range of biological scenarios. They argue that such broad applicability, without the need for manual processing of images, aids the analysis of situations typically beyond the range of quantitative measures developed for particular applications. For instance, they discuss using this measure to track changes in bilateral symmetry during growth, or even between species across evolutionary transitions. They primarily use examples generated from Turing mechanisms, but suggest that their proposed measure could aid in understanding the evolution of time-evolving symmetries, and hence give insight into possible mechanisms underlying symmetry breaking.

Gomez *et al.* [10] consider models of bulk and surface reaction–diffusion systems, which have become popular models for membrane-bound protein dynamics, among other applications. In addition to reviewing such systems and their mathematical analysis, they offer several additional perspectives, such as asymptotically deriving this class of models from suitably ‘thin’ outer regions, and relating these models to coupled partial and ordinary differential equations models of cell–cell interactions mediated over inactive bulk regions. They perform weakly nonlinear analysis, as well as far-from-equilibrium analysis of spike solutions, to demonstrate a range of dynamics which can be ascribed to the geometric coupling within these kinds of models. They highlight a range of open mathematical challenges, as well as opportunities for biological modelling using these frameworks. Veerman *et al*. [11] also discuss moving beyond classical reaction–diffusion systems. They describe the general ideas of Turing-like instabilities in abstract evolution equations, and how one can exploit scale separation via geometric singular perturbation theory to argue abstractly about the existence of patterned states in such systems, with some independence on the particular details of the model. They also describe alternatives to purely chemical frameworks, such as mechano-chemical models, which allow for pattern formation from quite different mathematical and physical scenarios. They discuss the relationship of their broad theoretical frameworks in the contexts of experimental observations, and suggest several novel research directions involving both modelling and rigorous mathematics, including the tuning or designing of systems with desired analytical properties.

Konow *et al*. [3] provide a comprehensive review of the theoretical and experimental literature on Turing patterns in chemical systems. Unlike the complexity and uncertainty of *in vivo* systems, chemical systems can exploit extremely well-characterized reaction kinetics and diffusion rates to design and engineer a range of pattern formation scenarios, from the interaction of coupled layers to growing domains using photosensitive reaction kinetics. They showcase several examples where this approach provides a window into fundamental aspects of these pattern-forming processes, where additional levels of complexity can be experimentally tuned (e.g. considering pattern formation in both static and growing domains, which is not often possible in developmental settings). They close their review by discussing future directions for chemical research in Turing-type patterning. Along similar lines, Vittadello *et al.* [12] discuss theoretical design principles in the engineering of synthetic Turing systems. They are motivated by recent efforts to engineer Turing type patterns *in vivo* using tools from synthetic biology. They review recent work in this area, discussing measures of robustness in the formulations of models, and how searching for robust networks in synthetic systems elucidates more general design principles. They argue that more effort should be spent on looking at larger classes of models, particularly when these can be engineered, in order to understand aspects of robustness observed in natural biological systems.

In conclusion, the papers in this theme issue provide an overview of the impact that Turing’s model (and extensions thereof) have had in biology, with an emphasis on the great deal of new mathematical theories and approaches that it has generated. While advances in biotechnology and computation have enabled us to make progress in several different directions and through multiple layers of complexity, importantly they highlight the need for more enhanced theoretical tools to interrogate new data. These articles highlight these issues, and summarize the current frontier of the theoretical landscape in Turing-type pattern formation. Lastly, they point to what are the key open problems and exciting challenges ahead, providing a roadmap for future development in this field.

## Data accessibility

This article has no additional data.
